# Supplementary material for: Retinoids stored locally in the lung are required to attenuate the severity of acute lung injury in male mice
Source: Nat Commun. 2023 Feb 15;14:851. doi: 10.1038/s41467-023-36475-3 (PMC9932169; doi:10.1038/s41467-023-36475-3)
Supplement: Supplementary file 1 — Supplementary Information [file 41467_2023_36475_MOESM1_ESM.pdf]

Retinoids Stored Locally in the Lung Are Required to Attenuate the Severity of Acute Lung Injury in Male Mice

Igor O. Shmarakov[1,2*], Galina A. Gusarova[1], Mohammad N. Islam[1], María Marhuenda-Muñoz[1,3,4], Jahar Bhattacharya[1], and William S. Blaner[1]

[1]Department of Medicine, Vagelos College of Physicians and Surgeons, Columbia University, New York, NY 10032, USA, [2]Department of Animal Sciences, School of Environmental and Biological Sciences, Rutgers, The State University of New Jersey, New Brunswick, NJ, 08901, USA, [3]Centro de Investigación Biomédica en Red Fisiopatología de la Obesidad y la Nutrición (CIBEROBN), Instituto de Salud Carlos III, 28029 Madrid, Spain and [4]Department of Nutrition, Food Science and Gastronomy, School of Pharmacy and Food Sciences and XIA, Institute of Nutrition and Food Safety (INSA-UB), University of Barcelona, 08921 Santa Coloma de Gramenet, Spain

*To whom correspondence should be addressed: Dr. Igor Shmarakov, Department of Animal Sciences, School of Environmental and Biological Sciences, Rutgers, The State University of New Jersey, Foran Hall 326, 59 Dudley Road, New Brunswick, NJ, 08901, USA.; Telephone 1-848-932-5617; E-mail: ishmarakov@sebs.rutgers.edu

Supplementary Figure 1.

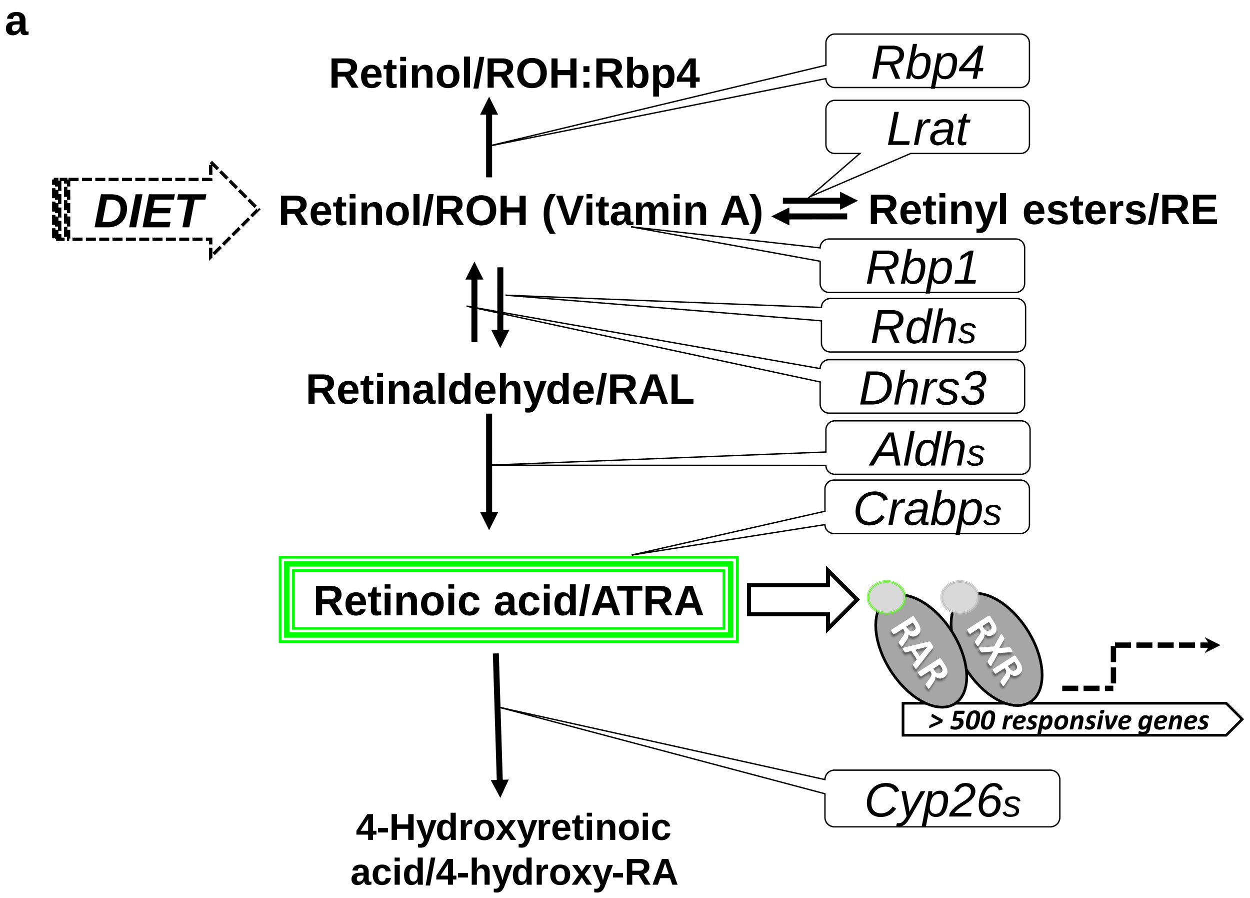

Supplementary Figure 1. Schematic representations of retinoid metabolism (panel a) and cellular diversity of alveolar cell types (panel b).

Panel a – Retinol (vitamin A) is acquired from the diet and can be enzymatically converted into different retinoid species. Retinyl esters (the predominant retinoid storage form) are synthesized from retinol via the action of lecithin:retinol acyltransferase (Lrat). Upon retinyl ester mobilization, retinol bound to retinol-binding protein 4 (Rbp4) can be secreted from the cell and transported throughout the body via the circulation to cells and tissues. Within the cell, retinol is bound to cellular retinol-binding protein 1 (Rbp1) and can be channeled towards oxidation to retinaldehyde via the action of retinol dehydrogenases (Rdhs). Retinaldehyde can be reversibly reduced to retinol via the action of short-chain dehydrogenase/reductase 3 (Dhrs3) or further oxidized by aldehyde dehydrogenases (Aldhs) to give rise to retinoic acid/ATRA, which can bind to cellular retinoic acid-binding proteins (Crabps). ATRA, the transcriptionally active retinoid species, binds to specific nuclear hormone receptors (Rars) which regulate the expression of more than 500 genes. Subsequently retinoic acid/ATRA undergoes oxidation to form inactive catabolic product 4-hydroxyretinoic acid via the action of specific cytochrome P450 isoforms (Cyp26s).

Supplementary Figure 1. (continued)

b

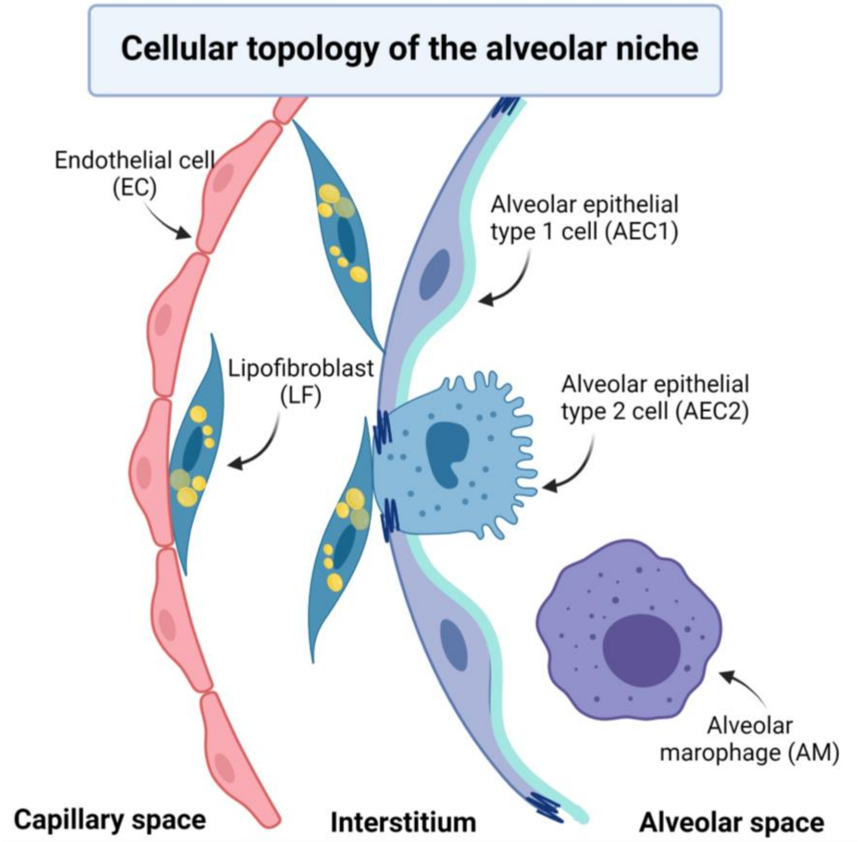

Supplementary Figure 1. Schematic representations of retinoid metabolism (panel a) and cellular diversity of alveolar cell types (panel b).

Panel b – The alveolus, the lung's functional unit where gas exchange occurs, is a structural and functional niche composed of heterogeneous cells of different origins. The major cells of the alveolar niche include endothelial cells that line microvascular blood capillaries, epithelial type 1 and type 2 cells that cover the area of the alveolar surface, fibroblasts that reside in the interstitium, an area around the wall of the alveolus through which oxygen moves from the alveoli into the capillary network, and alveolar macrophages. (Created with BioRender.com)

Supplementary Figure 2.

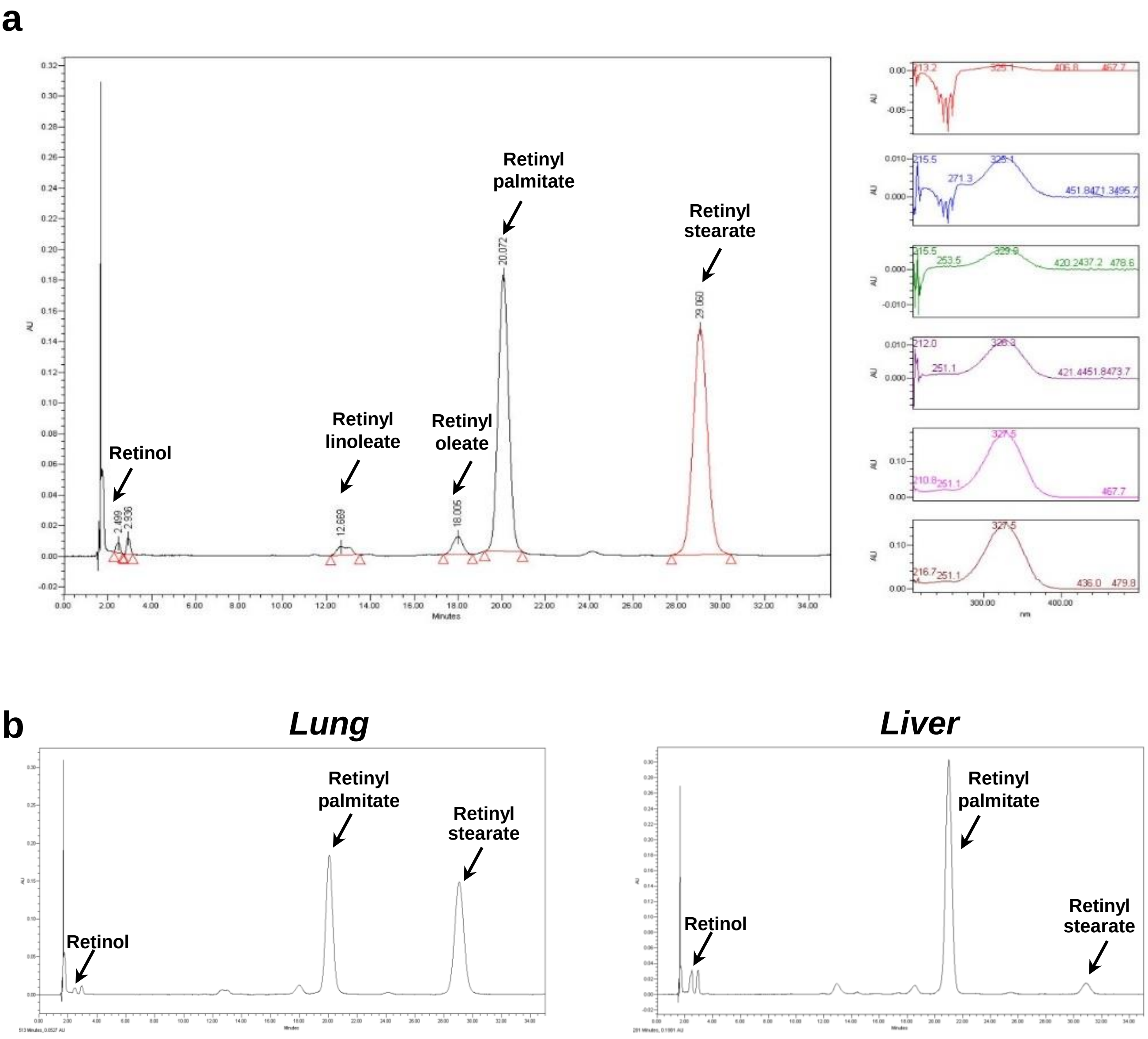

Supplementary Figure 2. Qualitative HPLC analysis of tissue retinoids.

Panel a – HPLC profile showing characteristic peaks of retinoid species (retinol and retinyl esters) extracted from a mouse lung homogenate; the right panel provides the absorption maxima for each peak, displaying a maximum at λ = 325 nm. ***Panel b*** – HPLC profiles of retinoid species (retinol and retinyl esters) extracted from lung and liver, showing the differences in retinyl ester compositions.

Supplementary Figure 3.

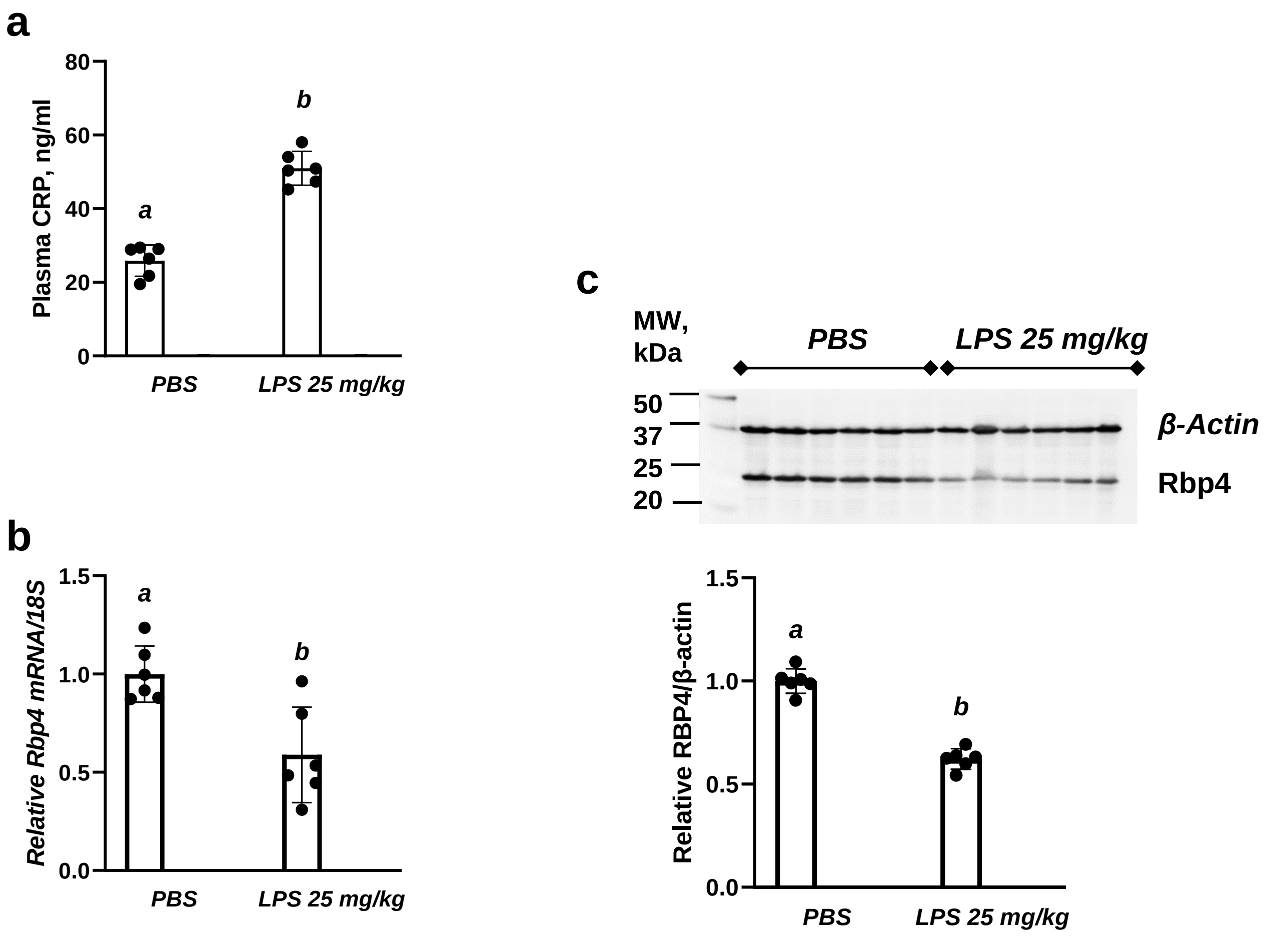

Supplementary Figure 3. Plasma C-reactive protein (Crp) concentration and hepatic Rbp4 mRNA and protein expression in mice upon LPS-induced ALI.

Panel a – C-reactive protein concentration (Crp) determined by ELISA in plasma of mice 7 days after intranasal instillation of LPS (25 mg/kg of body weight) or vehicle PBS alone. Values marked with different letters (a, b) are statistically different (a is different from b, p = 1.82e-06). Statistical differences were analyzed by a one-way ANOVA. All values are given as the mean ± 1 S.D., n = 6 for each group. ***Panel b*** – hepatic *Rbp4* mRNA expression determined by qRT-PCR in mice 7 days after intranasal instillation of LPS (25 mg/kg of body weight) or vehicle PBS. Values marked with different letters (a, b) are statistically different (a is different from b, p = 0.0051). Statistical differences were analyzed by a one-way ANOVA. All values are given as the mean ± 1 S.D., n = 6 for each group. ***Panel c*** – hepatic Rbp4 protein expression normalized to β-actin protein concentration determined by immunoblot (upper insert) in mice 7 days after intranasal instillation of LPS (25 mg/kg of body weight) or vehicle. Each lane represents an individual liver extract from a different mouse. Values marked with different letters (a, b) are statistically different (a is different from b, p = 3.12e-07). Statistical differences were analyzed by a one-way ANOVA. All values are given as the mean ± 1 S.D., n = 6 for each group.

Supplementary Figure 4.

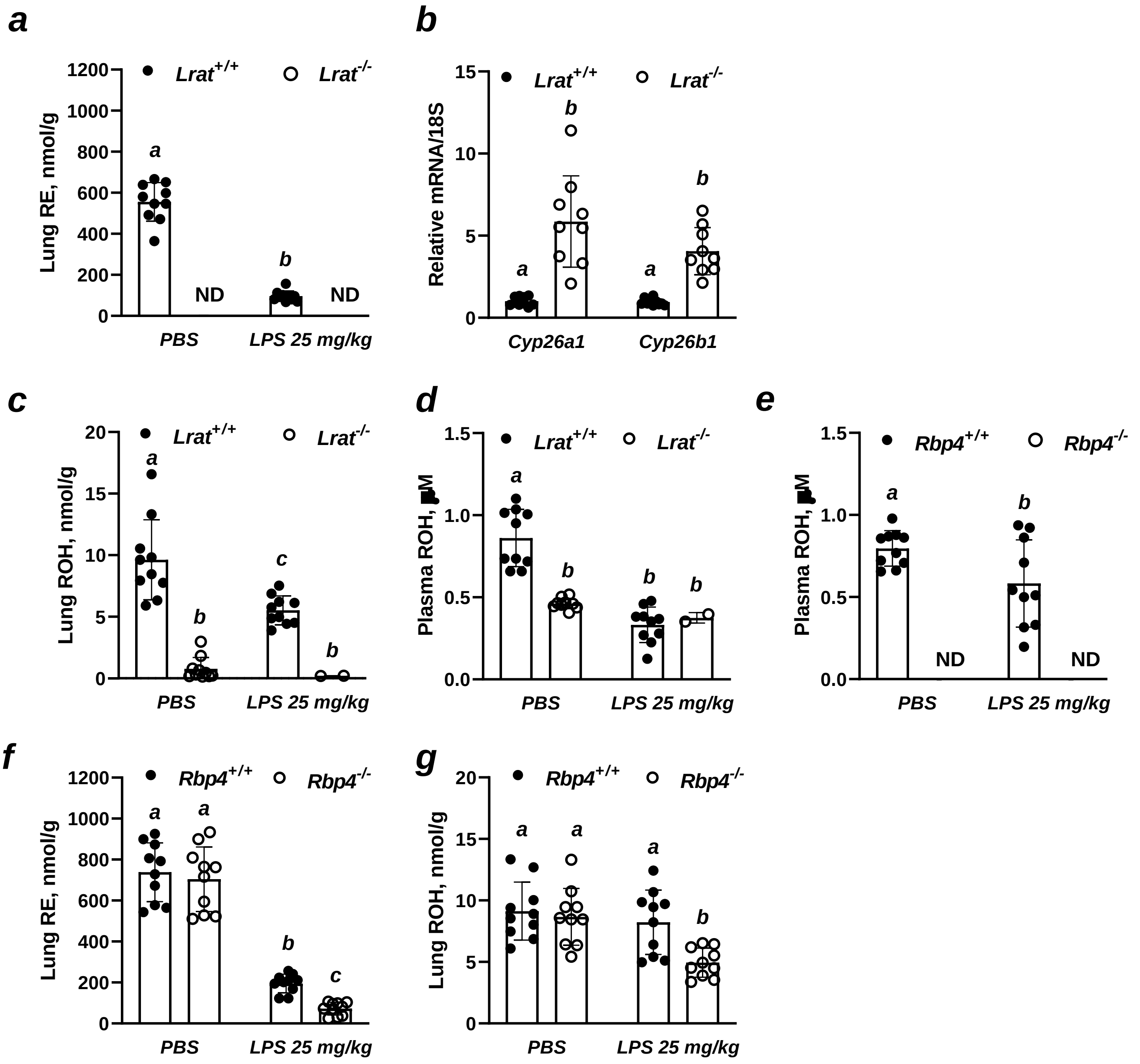

Supplementary Figure 4. Retinoid concentrations in mouse lungs and plasma during LPS-induced ALI.

Panel a – lung total retinyl ester (RE) concentrations (nmol/g) determined by HPLC in mice 7 days after intranasal instillation of LPS (25 mg/kg of body weight in PBS) or PBS alone. Values marked with different letters (a, b) are statistically different (a is different from b, p = 1.34e-11). Statistical differences were analyzed by a one-way ANOVA. All values are given as the mean ± 1 S.D., n = 10 for each group. ***Panel b*** – relative *Cyp26a1* and *Cyp26b1* mRNA expression (normalized to 18S rRNA levels) determined by qRT-PCR in lungs of *Lrat*$^{+/+}$ and *Lrat*$^{-/-}$ mice. Values marked with different letters (a, b) are statistically different (a is different from b, p = 3.82e-05 (for *Cyp26a1*), p = 3.19e-06 (for *Cyp26b1*)). Statistical differences were analyzed by a one-way ANOVA. All values are given as the mean ± 1 S.D., n = 10 for the *Lrat*$^{+/+}$ group, n = 9 for the *Lrat*$^{-/-}$ group. ***Panel c*** – lung retinol (ROH) concentrations (nmol/g) determined by HPLC in mice 7 days after intranasal instillation of LPS (25 mg/kg of body weight in PBS) or PBS alone. Values marked with different letters (a, b, c) are statistically different (a is different from b, p = 1.64e-10; a is different from c, p = 0.0001; b is different from c, p = 0.0021). Statistical differences were first analyzed by a one-way ANOVA followed by multiple comparisons employing Tukey's HSD post hoc test. All

Supplementary Figure 4. (continued)

values are given as the mean ± 1 S.D., n = 10 for each group, except for n = 2 for the LPS-treated *Lrat*$^{-/-}$ group (8 mice did not survive). ***Panel d –*** plasma retinol (ROH) concentrations (µM) determined by HPLC in mice 7 days after intranasal instillation of LPS (25 mg/kg of body weight in PBS) or PBS alone. Values marked with different letters (a, b) are statistically different (a is different from b, p = 3.07e-08). Statistical differences were first analyzed by a one-way ANOVA followed by multiple comparisons employing Tukey's HSD post hoc test. All values are given as the mean ± 1 S.D., n = 10 for each group, except for n = 2 for the LPS-treated *Lrat*$^{-/-}$ group (8 mice did not survive). ***Panel e*** – plasma retinol (ROH) concentrations (µM) determined by HPLC in mice 7 days after intranasal instillation of LPS (25 mg/kg of body weight in PBS) or PBS alone. Values marked with different letters (a, b) are statistically different (a is different from b, p = 0.03). Statistical differences were analyzed by a one-way ANOVA. All values are given as the mean ± 1 S.D., n = 10 for each group. ***Panel f*** – lung total retinyl ester (RE) concentrations (nmol/g) determined by HPLC in mice 7 days after intranasal instillation of LPS (25 mg/kg of body weight in PBS) or PBS alone. Values marked with different letters (a, b, c) are statistically different (a is different from b, p = 4.07e-13; a is different from c, p = 1.07e-15; b is different from c, p = 0.0172). Statistical differences were analyzed by a one-way ANOVA. All values are given as the mean ± 1 S.D., n = 10 for each group. ***Panel g*** – lung retinol (ROH) concentrations (nmol/g) determined by HPLC in mice 7 days after intranasal instillation of LPS (25 mg/kg of body weight in PBS) or PBS alone. Values marked with different letters (a, b) are statistically different (a is different from b, p = 0.0001). Statistical differences were first analyzed by a one-way ANOVA followed by multiple comparisons employing Tukey's HSD post hoc test. All values are given as the mean ± 1 S.D., n = 10 for each group.

Supplementary Figure 5.

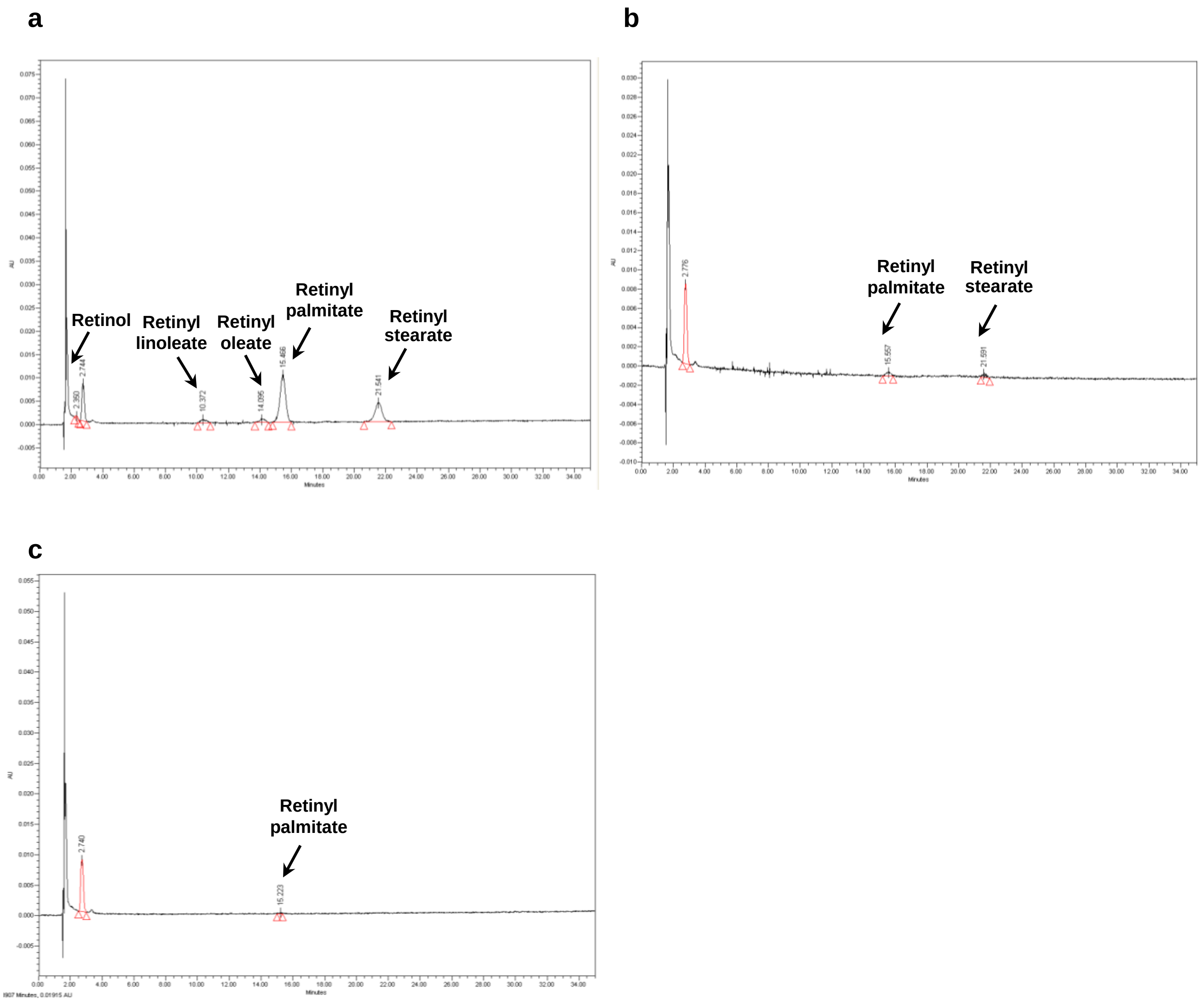

Supplementary Figure 5. Representative HPLC profiles for lung UV-positive cells isolated by FACS from a lung homogenate.

HPLC profiles showing characteristic UV-VIS absorption peaks for retinoid species (retinol and retinyl esters) extracted from isolated UV-positive cells (***panel a***), UV-positive endothelial cells (***panel b***), and UV-positive epithelial cells (***panel c***).

Supplementary Figure 6.

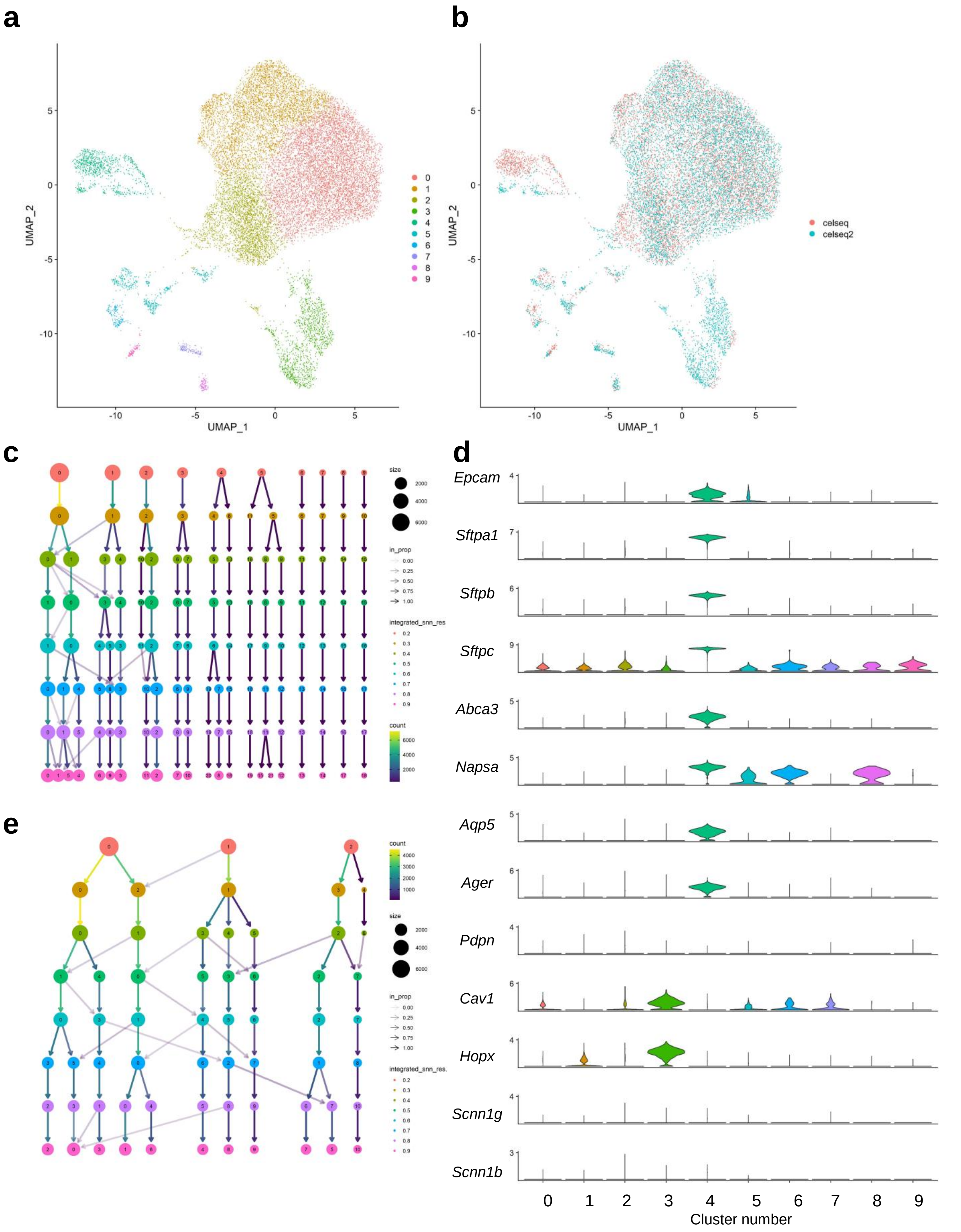

Supplementary Figure 6. Post-processing analysis of scRNA-seq data.

Panel a – UMAP visualization of cell type clustering inferred from mouse lung retinoid-containing cell scRNA-seq data; Ten retinoid-containing cell clusters were identified. ***Panel b*** – UMAP visualization of integrated batch-corrected scRNA-seq datasets colored by celseq

Supplementary Figure 6. (continued)

and celseq2 samples. ***Panel c*** – clustering tree of cell clusters with different clustering resolutions for scRNA-seq dataset of retinoid-containing lung cells. The size of each node is related to the number of cells in each cluster and the color indicates the clustering resolution; edges are colored according to the number of cells they represent and the transparency shows the incoming node proportion, the number of cells in the edge divided by the number of cells in the node it points to. ***Panel d*** – Violin plot representation showing expression of lung epithelial marker genes across all clusters. The y-axis indicates normalized Log2 expression value, the x-axis indicates cell cluster number. ***Panel e*** – clustering tree of cell clusters with different clustering resolutions for the scRNA-seq dataset of stromal retinoid-containing lung cells. The size of each node is related to the number of cells in each cluster and the color indicates the clustering resolution; edges are colored according to the number of cells they represent and the transparency shows the incoming node proportion, the number of cells in the edge divided by the number of cells in the node to which it points.

Supplementary Figure 7.

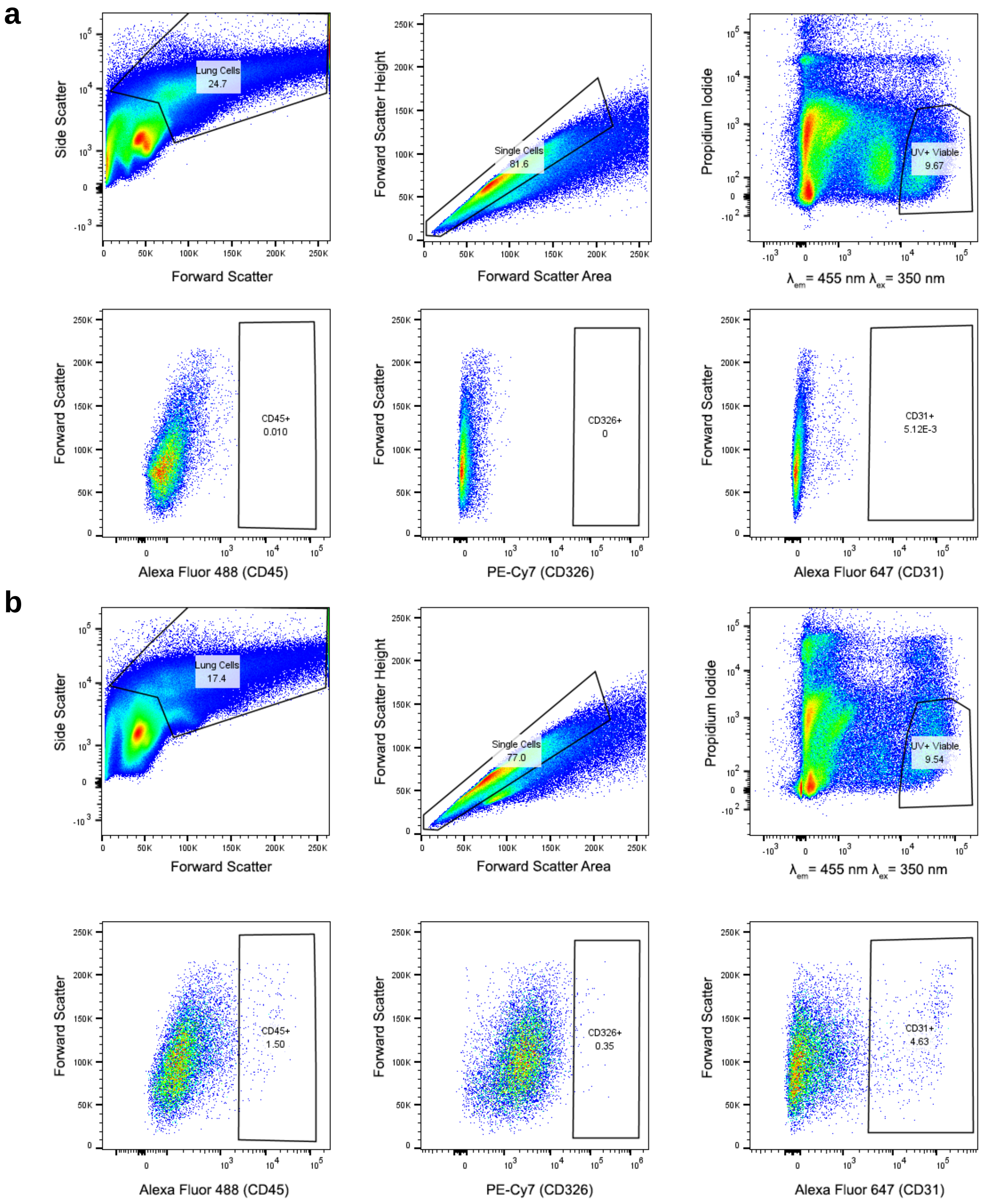

Supplementary Figure 7. FACS isolation and identification of lung retinoid-containing cells.
Panel a – A gating strategy for sorting live, single, UV-positive cells (emission at λ=455 nm upon excitation at λ=350 nm) from the lung cell suspensions isolated from *Lrat*$^{+/+}$ (wild type, C57BL/6) mice followed by the analysis of unstained cells for setting gating regions to discern positive from negative cells. ***Panel b*** – A gating strategy for sorting live, single, UV-positive cells (emission at λ=455 nm upon excitation at λ=350 nm) labeled with fluorochrome-conjugated antibodies to discern Cd45^{+} (immune), Cd326^{+} (epithelial), and Cd31^{+} (endothelial) cells from the lung cell suspensions isolated from *Lrat*$^{+/+}$ (wild type C57BL/6) mice. The numbers associated with each gate reflect the percentage of the parent population. Total events were gated for forward and side scatter signal to exclude debris, and then doublets were removed using a plot of forward scatter pulse area versus forward scatter pulse height. Cells containing retinoids were selected by measuring a blue (455 nm) signal at 350 nm excitation, and dead cells were removed through the exclusion of PI^{+} events. From this population, the percent of cells expressing Cd45, Cd326, and Cd31 were calculated and shown in each respective plot.

Supplementary Figure 8.

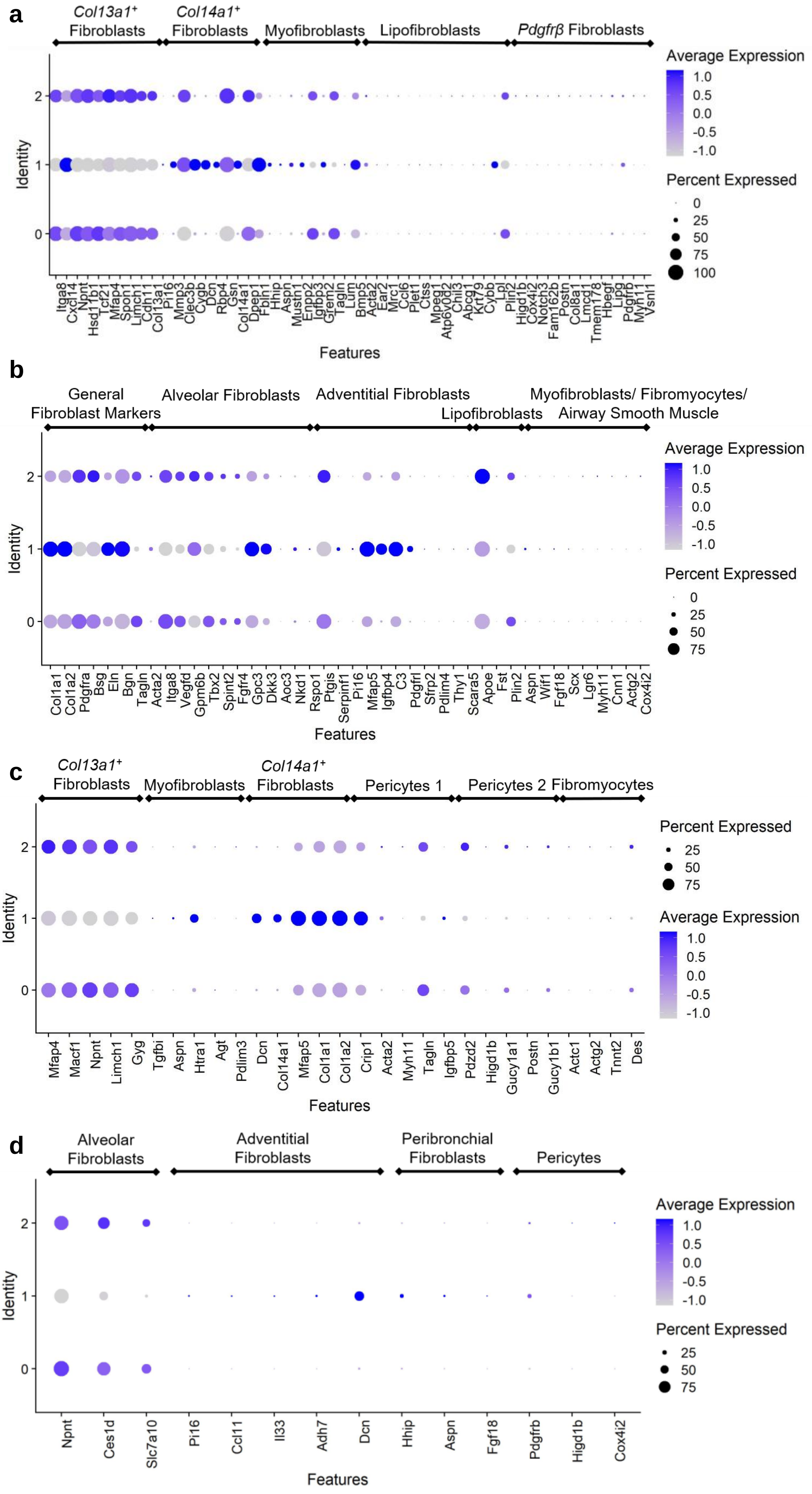

Supplementary Figure 8. (continued)

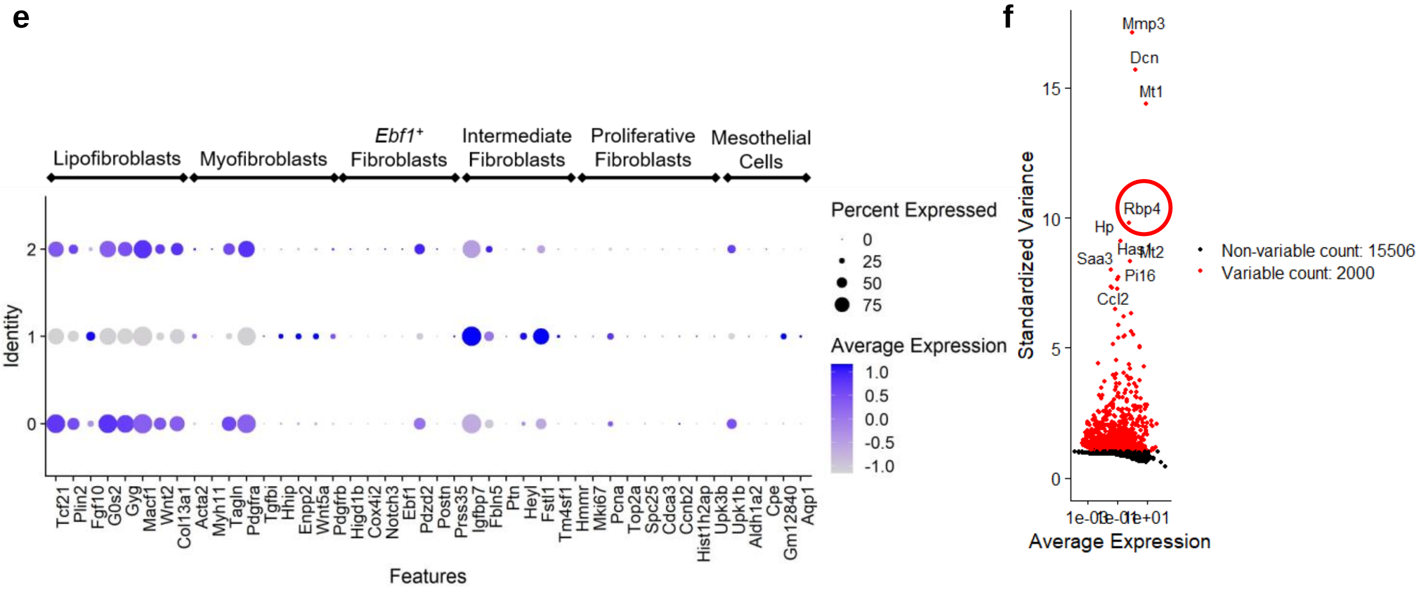

Supplementary Figure 8. Post-processing analysis and cell annotation of scRNA-seq data.

Panel a – Dot plot visualization of marker gene expression reported by Xie et al. [1] to annotate *Col13a1*$^+$ fibroblast, *Col14a1*$^+$ fibroblast, myofibroblast, lipofibroblast, and *Pdgfrβ* fibroblast clusters that was applied to stromal clusters from retinoid-containing cell scRNA-seq datasets generated in our study. ***Panel b*** – Dot plot visualization of marker gene expression reported by Travaglini et al. [2] to annotate general fibroblast, alveolar fibroblast, adventitial fibroblast, lipofibroblast, myofibroblast, fibromyocyte, and airway smooth muscle cell clusters that was applied to stromal clusters from retinoid-containing cell scRNA-seq datasets generated in our study. ***Panel c*** – Dot plot visualization of marker gene expression reported by Hurskainen et al. [3] to annotate *Col13a1*$^+$ fibroblast, *Col14a1*$^+$ fibroblast, myofibroblast, pericyte, and fibromyocyte clusters that was applied to stromal clusters from retinoid-containing cell scRNA-seq datasets generated in our study. ***Panel d*** – Dot plot visualization of marker gene expression reported by Tsukui et al. [4] to annotate alveolar fibroblast, adventitial fibroblast, peribronchial fibroblast, and pericyte clusters that was applied to stromal clusters from retinoid-containing cell scRNA-seq datasets generated in our study. ***Panel e*** – Dot plot visualization of marker gene expression reported by Liu et al. [5] to annotate alveolar lipofibroblast, myofibroblast, *Ebf1*$^+$ fibroblast, intermediate fibroblast, proliferative fibroblast, and mesothelial cell clusters that was applied to stromal clusters from retinoid-containing cell scRNA-seq datasets generated in our study. The x-axis (features) gives gene names, the y-axis (identity) indicates cluster numbers. The level of expression is specified by the color legend. Size of the cell population expressing the gene of interest is indicated by the size of the circle as specified by the legend. ***Panel f*** – Mean variability plot visualization of highly variable features (genes) identified in scRNA-seq datasets with *Rbp4* gene exhibiting one of the highest cell-to-cell variations in the whole dataset.

Supplementary Figure 9.

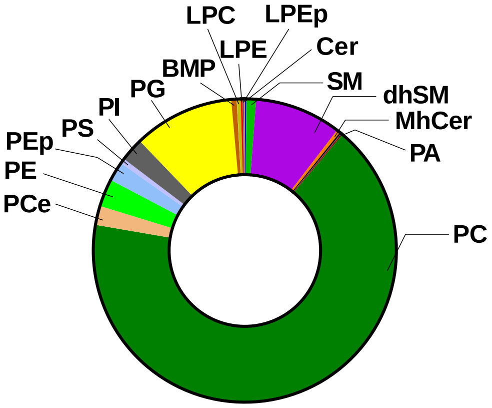

Lipid species	Mol %
Phosphatylcholine (PC)	66.87835
Phosphatidylglycerol (PG)	11.32748
Dihydrosphingomyelin (dhSM)	8.153958
Phosphatidylethanolamine (PE)	2.886557
Phosphatidylinositol (PI)	2.797658
Plasmalogen phosphatidylethanolamine (Pep)	2.154491
Ether phosphatidylcholine (PCe)	2.058167
Sphingomyelin (SM)	1.015562
Bis(monoacylglycero)phosphate (BMP)	0.591869
Phosphatidylserine (PS)	0.503343
Monohexosylceramide (MhCer)	0.364123
Lysophosphatidylcholine (LPC)	0.326848
Lysophosphatidylethanolamine (LPE)	0.176749
Sulfatide (Sulf)	0.161722
Ceramide (Cer)	0.140697
Phosphatidic acid (PA)	0.140075
Plasmogen Lysophosphatidylethanolamine (LPEp)	0.096730
Lysophosphatidylinositol (LPI)	0.077462
Lysophosphatidylserine (LPS)	0.061962
Acyl Phosphatidylglycerol (AcylPG)	0.022806
Lactosylceramide (LacCer)	0.022361
Monosialodihexosylganglioside (GM3)	0.019581
Ether lysophosphatidylcholine (LPCe)	0.005497
Dihydroceramide (dhCer)	0.004984
N-Acyl Phosphatidylethanolamine (NAPE)	0.004202
N-Acyl Serine (NSer)	0.003848
Globotriaosylceramide (GB3)	0.002660
N-Acyl Phosphatidylserine (NAPS)	0.000251

Supplementary Figure 9. Mouse BAL fluid lipid composition.

Lipid species detected by UPLC-MS/MS and their mol % in mouse BAL fluid.

Supplementary Figure 10.

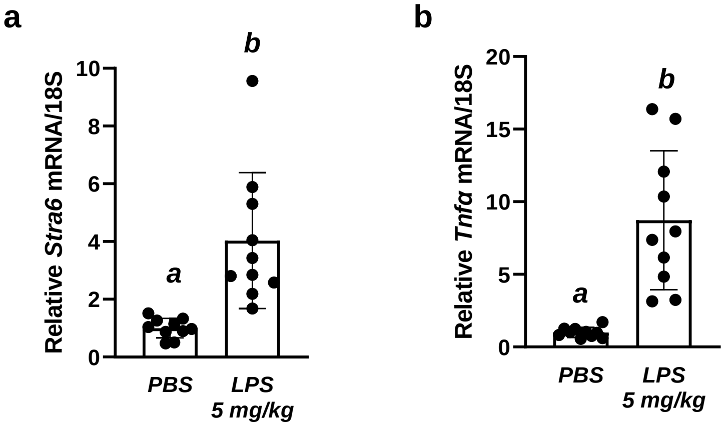

Supplementary Figure 10. Relative mRNA expression of *Stra6* (panel a) and *Tnfα* (panel b) in cells isolated from BAL fluid of mice after LPS instillation.

Gene expression values (normalized to 18S rRNA levels) were determined by qRT-PCR. Values marked with different letters (a, b) are statistically different (a is different from b, p = 0.00079 (for *Stra6*), p = 7.72e-05 (for *Tnfα*). Statistical differences were analyzed by a one-way ANOVA. All values are given as the mean ± 1 S.D., n = 10 for each group.

Supplementary Figure 11.

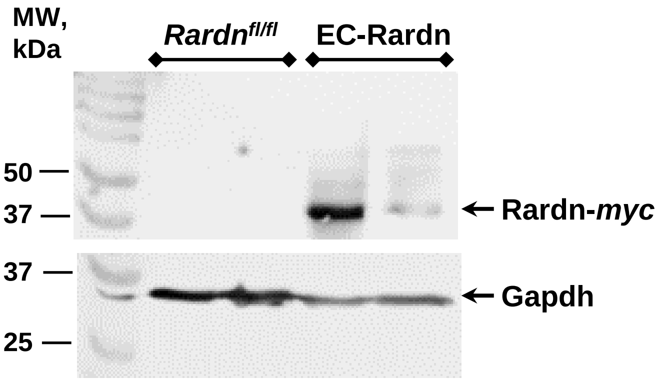

Supplementary Figure 11. Rardn protein expression in isolated lung endothelial cell.

Lung endothelial cell Rardn protein expression (c-*myc*-tagged protein) normalized to Gapdh protein concentration determined by immunoblot in EC-Rardn and Rardn$^{fl/fl}$ mice one month after tamoxifen administration. Each lane represents an individual endothelial cell extract from a different mouse. Similar results were reproduced in three independent experiments.

Supplementary Table 1.

Antibodies used for flow cytometric analysis

Antigen	Clone	Laser	Fluorochrome	Manufacturer
Cd45	30-F11	488	Alexa Fluor488	Biolegend
Cd326	G8.8	561	PE-Cy7	Biolegend
Cd31	390	637	Alexa Fluor 647	Biolegend

Supplementary Table 2.

Primer sequences used for qRT-PCR

Gene name	Accession number	Primers (forward/reverse)
18S		5'-CCATCCAATCGGTAGTAGCG
		5'-GTAACCCGTTGAACCCCATT
Lrat	NM_023624	5'-AGTTCAAGACTAGCCTGCTCA
		5'-TACAAGCTGGCCTTCGAC
Rbp1	NM_011254	5'-AGAAGACAGAACCACACGA
		5'-GGTGGTCAGAGACCCAAG
Aldh1a1	NM_001361503	5'-TGTTGAGGAGTCAGTTTATGATG
		5'-GGCTCCTTCTTTCTTCCC
Rdh10	NM_133832	5'-TCTACCGGGACCTGGAGGC
		5'-TCCAGAAACCACACCAGCA
Cyp26a1	NM_007811	5'-AGAGCAATCAAGACAACAAGTTAG
		5'-ATCGCAGGGTCTCCTTAAT
Cyp26b1	NM_175475	5'-TCTCTGCCAGGTGTACTTAG
		5'-CCTTCAGAAGAACCCGTATC
Rarα	NM_009024	5'-AATCTGCACGCGGTACA
		5'-TCAGCATCGTCCATCTCC
Rarβ	NM_011243	5'-CAAGTTCAAGTGGGAATATAGCAGA
		5'-ACTGACTGACTCCACTGTT
Rarγ	NM_011244	5'-CAGAGGGTCGCCACCATT
		5'-ATCTCCTCCGAGCTGGTGC
Rbp4	NM_001159487	5'-GAGTCCGTCTTCTGAGCAAC
		5'-CTTGAACTTGGCAGGATCT
Stra6	NM_001162476	5'-CCTGGGCCTTCTCCCATCAT
		5'-CCTGGTAAGTGGCTGTTCCTGTCA
Lpl	NM_008509	5'-GCTGGTGGGAAATGATGT
		5'-ACCAGTAATTCTATTGACCTTCTTAT
Cd36	NM_001159558	5'-GCTGTGTTTGGAGGCATTCT
		5'-CCTTGATTTTGCTGCTGTTC
Scarb1	NM_016741	5'-CCCAAACGAGGTCCTCA
		5'-ACTTGTCAGGCTGGAAAT
Sftpb	NM_147779	5'-CCCAGCTCTAACTACAGAC
		5'-CCCTTCTGAAGGCTTCCA
Sftpc	NM_011359	5'-TGATGGAGAGTCCACCGGAT
		5'-CCACCACAACCACGATGAGA
Cdh1	NM_009864	5'-GGTCTTTCAGCTCCTTCC
		5'-AGGCACAGTTTATATCTCAGCA
Cdh5	NM_009868	5'-TTATTATAAGACTCTAGTTCTCACAGACA
		5'-CATTATCCTTACAGCAATGACTAC
Ocln	NM_001360536	5'-CTGACCTTGAGTGTGGA
		5'-CACCTGTCGTGTAGTCTG
Tjp1	NM_009386	5'-GGTCAAATGAAGACAATTACTTATTGTAT
		5'-CCCACTAGGGTAAGGCA

Supplementary References:

1. Xie, T. *et al.* Single-Cell Deconvolution of Fibroblast Heterogeneity in Mouse Pulmonary Fibrosis. *Cell Rep* **22**, 3625-3640, doi:10.1016/j.celrep.2018.03.010 (2018).
2. Travaglini, K. J. *et al.* A molecular cell atlas of the human lung from single-cell RNA sequencing. *Nature* **587**, 619-625, doi:10.1038/s41586-020-2922-4 (2020).
3 Hurskainen, M. *et al.* Single cell transcriptomic analysis of murine lung development on hyperoxia-induced damage. *Nat Commun* **12**, 1565, doi:10.1038/s41467-021-21865-2 (2021).
4. Tsukui, T. *et al.* Collagen-producing lung cell atlas identifies multiple subsets with distinct localization and relevance to fibrosis. *Nat Commun* **11**, 1920, doi:10.1038/s41467-020-15647-5 (2020).
5. Liu, X. *et al.* Categorization of lung mesenchymal cells in development and fibrosis. *iScience* **24**, 102551, doi:10.1016/j.isci.2021.102551 (2021).
