# Supplementary material for: Retinoids stored locally in the lung are required to attenuate the severity of acute lung injury in male mice
Source: Nat Commun. 2023 Feb 15;14:851. doi: 10.1038/s41467-023-36475-3 (PMC9932169; doi:10.1038/s41467-023-36475-3)
Supplement: Supplementary file 3 — Reporting Summary [file 41467_2023_36475_MOESM3_ESM.pdf]

nature portfolio

Corresponding author(s): Igor Shmarakov

Last updated by author(s): Jan 23, 2023

Reporting Summary

Nature Portfolio wishes to improve the reproducibility of the work that we publish. This form provides structure for consistency and transparency in reporting. For further information on Nature Portfolio policies, see our Editorial Policies and the Editorial Policy Checklist.

Statistics

For all statistical analyses, confirm that the following items are present in the figure legend, table legend, main text, or Methods section.

n/a	Confirmed	
☐	☒	The exact sample size (*n*) for each experimental group/condition, given as a discrete number and unit of measurement
☐	☒	A statement on whether measurements were taken from distinct samples or whether the same sample was measured repeatedly
☐	☒	The statistical test(s) used AND whether they are one- or two-sided<br>*Only common tests should be described solely by name; describe more complex techniques in the Methods section.*
☒	☐	A description of all covariates tested
☒	☐	A description of any assumptions or corrections, such as tests of normality and adjustment for multiple comparisons
☐	☒	A full description of the statistical parameters including central tendency (e.g. means) or other basic estimates (e.g. regression coefficient) AND variation (e.g. standard deviation) or associated estimates of uncertainty (e.g. confidence intervals)
☐	☒	For null hypothesis testing, the test statistic (e.g. *F*, *t*, *r*) with confidence intervals, effect sizes, degrees of freedom and *P* value noted<br>*Give P values as exact values whenever suitable.*
☒	☐	For Bayesian analysis, information on the choice of priors and Markov chain Monte Carlo settings
☒	☐	For hierarchical and complex designs, identification of the appropriate level for tests and full reporting of outcomes
☒	☐	Estimates of effect sizes (e.g. Cohen's *d*, Pearson's *r*), indicating how they were calculated

Our web collection on statistics for biologists contains articles on many of the points above.

Software and code

Policy information about availability of computer code

Data collection	No software was used for data collection.
Data analysis	Empower v2, Masshunter v7.0, FlowJo v10, Cell Ranger v5.0.1, R v4.1.1, Seurat v4.0.4, LightCycler 480 v1.5.0.39, LI-COR Acquisition Software v1.0.19, Image Studio v5.2, ImageJ v1.53a, GraphPad Prism v9. Code used for the analysis of scRNA-seq data is available at the public GitHub repository at https://github.com/ishmarakov/scRNAseq_lung_retinoid

For manuscripts utilizing custom algorithms or software that are central to the research but not yet described in published literature, software must be made available to editors and reviewers. We strongly encourage code deposition in a community repository (e.g. GitHub). See the Nature Portfolio guidelines for submitting code & software for further information.

Data

Policy information about availability of data

All manuscripts must include a data availability statement. This statement should provide the following information, where applicable:

- Accession codes, unique identifiers, or web links for publicly available datasets
- A description of any restrictions on data availability
- For clinical datasets or third party data, please ensure that the statement adheres to our policy

The scRNA-seq data generated in this study are deposited in Gene Expression Omnibus (GEO) under accession number GSE198521. The source data underlying the figures are provided as a Source Data file.

Human research participants

Policy information about studies involving human research participants and Sex and Gender in Research.

Reporting on sex and gender	N/A
Population characteristics	N/A
Recruitment	N/A
Ethics oversight	N/A

Note that full information on the approval of the study protocol must also be provided in the manuscript.

Field-specific reporting

Please select the one below that is the best fit for your research. If you are not sure, read the appropriate sections before making your selection.

☒ Life sciences ☐ Behavioural & social sciences ☐ Ecological, evolutionary & environmental sciences

For a reference copy of the document with all sections, see nature.com/documents/nr-reporting-summary-flat.pdf

Life sciences study design

All studies must disclose on these points even when the disclosure is negative.

Sample size	Sample sizes were based on the experience of the authors with molecular and in vivo studies as published in many studies. For animal models, experiments were designed to detect differences between treatment groups or genotype-dependent effects at 80% power (α=0.05).
Data exclusions	For scRNA-seq post processing analysis, cells with fewer than 200 detected genes or more than 10 % mitochondrial genes were excluded. These are pre-established exclusion criteria for removing low quality cells from the analysis.
Replication	All experiments were replicated at least twice and on different occasions.
Randomization	Mice were randomly assigned to each group by simple randomization
Blinding	For scRNA-seq, researcher performing the sequencing was blinded to data collection. Data analysis of scRNA-seq was not blinded, as the analysis is entirely data driven and therefore unbiased. Blinding was not possible in the majority of the wet lab experiments because they were done by one single person. For HPLC, UPLC-MS/MS, qRT-PCR, and western blotting, researchers performing these analyses were blinded to sample allocation.

Reporting for specific materials, systems and methods

We require information from authors about some types of materials, experimental systems and methods used in many studies. Here, indicate whether each material, system or method listed is relevant to your study. If you are not sure if a list item applies to your research, read the appropriate section before selecting a response.

Materials & experimental systems

n/a	Involved in the study	
☐	☒	Antibodies
☒	☐	Eukaryotic cell lines
☒	☐	Palaeontology and archaeology
☐	☒	Animals and other organisms
☒	☐	Clinical data
☒	☐	Dual use research of concern

Methods

n/a	Involved in the study	
☒	☐	ChIP-seq
☐	☒	Flow cytometry
☒	☐	MRI-based neuroimaging

Antibodies

Antibodies used	Primary rabbit polyclonal antibody against mouse retinol-binding protein 4 (in-house generated, 1:3,000), primary rabbit polyclonal antibody against mouse lecithin:retinol acyltransferase (Novus Biologicals, cat.# NBP3-04496, 1:2,000), primary mouse monoclonal antibody against synthetic myc peptide (Invitrogen, cat.# MA1-21316-HRP, clone Myc.A7, 1:2,000), primary rabbit monoclonal antibody against Gapdh (Cell Signaling, cat.# 2118, 1:1,000), primary mouse monoclonal antibody against β-actin (Millipore Sigma,

	cat.# A2228, clone AC-74, 1:10,000). Secondary donkey anti-rabbit antibody (ThermoFisher Scientific, cat.# 31458) and sheep anti-mouse antibody (GE Healthcare, cat.# NA931). Alexa Fluor 647 anti-mouse Cd31 antibody (Biolegend, cat#102415, lot# B357367, 1:200), Alexa Fluor 488 anti-mouse Cd45 antibody (Biolegend, cat#103121, lot#B285086, 1:200), PE/Cyanine7 anti-mouse Cd326 antibody (Biolegend, cat#118215, lot#B361565).
Validation	In-house generated anti-RBP4 antibody was validated using purified RB4 protein and plasma samples from Rbp4 knockout mice. Commercially available antibodies were validated by manufacturers to react with mouse proteins. Manufacturer's website containing validation data for the commercially available antibodies and citations for the custom-made antibodies are listed below:<br>1. Rabbit polyclonal antibody against mouse lecithin:retinol acyltransferase (Novus Biologicals, cat.# NBP3-04496) https://www.novusbio.com/products/lrat-antibody_nbp3-04496#reviews-publications.<br>2. Mouse monoclonal antibody against synthetic myc peptide (Invitrogen, cat.# MA1-21316-HRP, clone Myc.A7) https://www.thermofisher.com/antibody/product/Myc-Tag-Antibody-clone-Myc-A7-Monoclonal/MA1-21316-HRP<br>3. Rabbit monoclonal antibody against Gapdh (Cell Signaling, cat.# 2118, 1:1,000) https://www.cellsignal.com/products/primary-antibodies/gapdh-14c10-rabbit-mab/2118<br>4. Mouse monoclonal antibody against β-actin (Millipore Sigma, cat.# A2228, clone AC-74) https://www.sigmaaldrich.com/US/en/product/sigma/a2228<br>5. Alexa Fluor 647 anti-mouse Cd31 antibody (Biolegend, cat#102415, lot# B357367) https://www.biolegend.com/en-us/clone-search/alexa-fluor-647-anti-mouse-cd31-antibody-3092?GroupID=BLG1566<br>6. Alexa Fluor 488 anti-mouse Cd45 antibody (Biolegend, cat#103121, lot#B285086) https://www.biolegend.com/en-us/punchout/punchout-products/product-detail/alexa-fluor-488-anti-mouse-cd45-antibody-3100?GroupID=BLG1932<br>7. PE/Cyanine7 anti-mouse Cd326 antibody (Biolegend, cat#118215, lot#B361565) https://www.biolegend.com/en-us/punchout/punchout-products/product-detail/pe-cyanine7-anti-mouse-cd326-ep-cam-antibody-5303

Animals and other research organisms

Policy information about studies involving animals; ARRIVE guidelines recommended for reporting animal research, and Sex and Gender in Research

Laboratory animals	Three-month-old (10-12 weeks-old) male mice were used in all studies. Specific strains of mice that were used in the experiments were on C57BL/6 or C57BL/6J/129 SV mixed genetic background.
Wild animals	No wild animals were used
Reporting on sex	Groups of male mice were used in all studies.
Field-collected samples	No field-collected samples were used
Ethics oversight	All experiments involving mice were carried out with the approval of the Institutional Animal Care and Use Committee of Columbia University according to criteria outlined in the Guide for the Care and Use of Laboratory Animals prepared by the National Academy of Sciences

Note that full information on the approval of the study protocol must also be provided in the manuscript.

Flow Cytometry

Plots

Confirm that:

- [x] The axis labels state the marker and fluorochrome used (e.g. CD4-FITC).
- [x] The axis scales are clearly visible. Include numbers along axes only for bottom left plot of group (a 'group' is an analysis of identical markers).
- [x] All plots are contour plots with outliers or pseudocolor plots.
- [x] A numerical value for number of cells or percentage (with statistics) is provided.

Methodology

Sample preparation	Mouse lung digests for single-cell flow cytometry were prepared from wild type C57BL/6 mice using a method involving digestion with pronase and collagenase. The lungs of anesthetized mice were first perfused through the right ventricle with calcium- and magnesium-free Hanks' balanced salt solution (HBSS). Then the lungs were perfused in situ with HBSS containing calcium, magnesium, and Dispase II (Sigma-Aldrich) followed by a second perfusion with HBSS containing calcium, magnesium, and type IV collagenase (Worthington). The lungs were removed, rinsed and minced into small pieces. The minced lung tissue was incubated in HBSS containing Dispase II (Sigma-Aldrich), type IV collagenase (Worthington), and DNase I (Sigma-Aldrich) on a rotating shaker maintained at 37 °C for 60 min. At each of three 20-min intervals, the minced tissue was then passed 10-times through a 10-ml pipette to dissociate cells. The resultant cell suspension was next passed through a 100 μm strainer to collect single cells and an equal volume of cold (4°C) complete medium containing DMEM, 10% fetal bovine serum (FBS), penicillin and streptomycin (10,000 units/100 ml) was added. Cells were pelleted by centrifugation at 500 X g for 10 min and resuspended in DMEM containing 10% FBS.<br>For flow cytometry isolations, cells were incubated with FcBlock (Biolegend) and stained with a mixture of fluorochrome-conjugated antibodies.

Instrument	FACSAria cell sorter (Becton Dickinson)
Software	FlowJo v10 (TreeStar, Ashland, OR)
Cell population abundance	Post-sort purity was checked by re-analyzing the sorted cells; the purity was more than 90 %.
Gating strategy	Flow cytometry was employed to sort live, individual retinoid-containing cells based on their emission at 455 nm (UV-positive cells). Singlet discrimination was sequentially performed using plots for forward scatter (FSC-A versus FSC-H) and side scatter (SSC-W versus SSC-H). Dead cells were excluded by scatter characteristics and staining with propidium iodide. Among UV-positive cells, subsets of cells with high expression of Cd45 (immune cells), Cd326 (epithelial cells), and Cd31 (endothelial cells) were identified, separated, and collected for further analysis.

☒ Tick this box to confirm that a figure exemplifying the gating strategy is provided in the Supplementary Information.
